# Multidisciplinary Telehealth for Pediatric Developmental and Mental Health Care in Rural and Remote Australia: Interview Study Among Caregivers and Service Providers

**DOI:** 10.2196/89927

**Published:** 2026-07-23

**Authors:** Leda Sivak, Marcel Zimmet, Blake Dear, Rosemary Evans, Jacqueline Emery, Allison Waters, Elizabeth J Elliott

**Affiliations:** 1 Griffith University, Gold Coast, Queensland, Australia; 2 Royal Far West, 14-18 Wentworth Street, Manly, New South Wales, 2095, Australia, +61289668500; 3 Macquarie University, Sydney, New South Wales, Australia; 4 The University of Sydney, Sydney, New South Wales, Australia

**Keywords:** rural, remote, developmental, pediatric, qualitative

## Abstract

**Background:**

Geography and socioeconomic disadvantage impede access to appropriate services for rural and remote-dwelling children with complex developmental and mental health needs. Telehealth offers opportunities to increase access to essential health and well-being services for people with complex needs who live in rural and remote areas, whose experiences of mental ill-health are frequently compounded by social marginalization and economic disadvantage.

**Objective:**

This study aimed to describe caregivers’ and service providers’ perspectives on the challenges and value of telehealth services for rural and remote children by drawing on experiences within a specialist service (Royal Far West; RFW) that provides mental and developmental health services to rural and remote-dwelling children and families in Australia.

**Methods:**

Participants included a convenience sample of caregivers of children who use RFW services (n=11) and service providers from RFW (n=21). Deidentified interview transcript data were analyzed thematically using framework analysis.

**Results:**

Caregivers were generally enthusiastic about telehealth but acknowledged potential technological limitations and challenges to effectively engaging some children. While acknowledging its value and importance, RFW service providers identified additional challenges with providing telehealth, such as limited capacity for some detailed clinical assessments and potential child and family risk and safety issues.

**Conclusions:**

Telehealth remains a vital option within multidisciplinary services to improve access and outcomes for children in rural and remote communities who present with complex mental and developmental health needs. Despite challenges, rural and remote caregivers and RFW service providers identified telehealth as a beneficial, accessible, and effective component of multidisciplinary care for children with complex mental and developmental health needs. These caregiver and service provider perspectives will inform ongoing service development, research, and advocacy.

## Introduction

### Background

Geography and socioeconomic disadvantage often make it difficult for rural and remote-dwelling Australian children with complex developmental and mental health needs to access appropriate services [1]. Clinical, family, and community complexity likely also influences service engagement for these families, whose experiences of mental ill-health are frequently compounded by social marginalization and economic disadvantage [2]. Although the prevalence of child and youth mental health problems is increasing across Australia [3], fewer than one-third of children currently receive the mental health services they require nationally [4]. Furthermore, although children and young people living in rural and remote communities experience poorer mental health [5] and developmental outcomes [6] than those in urban locations, their use of mental health services is considerably lower than that in urban areas in Australia [7] and internationally [8,9].

Telehealth offers opportunities to increase access to essential health and well-being services for children with complex mental and developmental health needs who live in rural and remote areas [10]. In this paper, “telehealth” refers to a combination of video consultations, telephone calls, and digital platforms used for client engagement, assessment, and therapy, which are sometimes supplemented with face-to-face consultations. A recent systematic review of telehealth screening, assessment, and diagnosis of autism spectrum disorder (ASD) found that telehealth facilitated the standardization, sensitivity, specificity, and efficiency of screening, assessment, and diagnosis of ASD [11]. Similarly, a systematic review of telehealth services to assess and treat neurodevelopmental disorders found that these services were clinically effective, cost-effective, and acceptable to caregivers [12]. Telehealth has been found to be convenient and cost-effective, as well as supportive for high-risk populations, given its ability to remove geolocation barriers [13]. Furthermore, telehealth as a psychological intervention for caregivers of children with neurodevelopmental disorders has been shown to improve mental health outcomes [14].

Telehealth is also acceptable to children and families seeking diverse and complex health and well-being care remotely [15]. For example, a recent systematic review of telehealth interventions for children with ASD found that telehealth programs are highly acceptable even when compared to face-to-face interventions and noted the vital role of telehealth for enhancing equitable access to interventions for rural and remote communities [16]. When facilitated through partnerships with Aboriginal community-controlled health services, telehealth services are also effective and acceptable to Aboriginal and Torres Strait Islander individuals and communities [17]. Furthermore, ease of use, improvements in outcomes and communication, and reductions in costs support patient satisfaction with telehealth [18].

However, most pediatric neurodevelopmental studies from Australia and abroad have reported telehealth benefits primarily in urban populations, providing limited data on rural and remote communities. Furthermore, they have not explored hybrid telehealth and face-to-face models. There is a need for studies focused on these populations and models of care to address knowledge gaps. Therefore, the aim of this study was to describe caregivers’ and service providers’ perspectives on the challenges and value of telehealth services by drawing on experiences within Royal Far West (RFW), a specialist service providing mental and developmental health services to rural and remote-dwelling children and families in Australia.

### Study Context

RFW is a charitable, nongovernment, specialist child health service located in Sydney, New South Wales, Australia, that provides developmental, mental health, and disability support for children up to the age of 12 years in rural and remote settings using a mixed model of care that includes telehealth. Given its clientele, RFW was an early adopter of telehealth, using it for assessment and therapy in a range of clinical disciplines since 2015, well before the COVID-19 pandemic accelerated its use. RFW services have been adapted over time to integrate telehealth within their standard models of care, with telehealth becoming an integral modality to enhance and maintain engagement and reduce barriers to access.

Thus, telehealth is part of the standard assessment and therapy provided by RFW in all clinical services. Telehealth at RFW is provided by speech pathologists, occupational therapists, psychologists, social workers, nurses, pediatricians, and psychiatrists. It is used as both stand-alone care and to complement face-to-face care. For example, in the assessment-focused pediatric developmental service, telehealth is used before, after, and between face-to-face residential assessment visits in Sydney. Telehealth introduction consultations involve a pediatrician and allied health clinician connecting with the child and their caregivers, reviewing referral information, and assisting in planning for the upcoming visit. After the on-site or in-community (outreach) assessment with a multidisciplinary team, telehealth is used for diagnostic feedback from the multidisciplinary team and care planning with the caregiver. It is also used for ongoing care (eg, for medication management), interim progress reviews prior to further face-to-face assessments, and group parenting programs. Telehealth sessions are typically conducted with the child and caregiver at home, although for families with limited internet connectivity or other problems with access, sessions are conducted from the child’s school or another service provider (eg, child protection or health service offices).

RFW’s community-based disaster recovery service provides individual telehealth therapy sessions to children impacted by bushfires and floods. Telehealth is used for therapy (eg, eight 45-minute weekly sessions for psychological, occupational, or speech therapy) or for parent capacity building (eg, *Tuning in to Kids* parenting program). These programs supplement children’s group programs or teacher capacity building programs that are delivered face-to-face during in-community (outreach) visits. Thus, at RFW, telehealth is integrated into all models of care and used for rapport building and service familiarization, diagnostic assessment, feedback and psychoeducation, therapy, care planning, progress review, and follow-up, as well as parenting programs, often to complement face-to-face care. Hence, telehealth can be an enabler of service engagement for rural and remote families along their care journey when used dynamically.

## Methods

### Study Design

The data presented in this paper were collected as part of a broader exploratory sequential mixed methods study. For the qualitative component of the study, a convenience sample of current RFW service providers and caregivers of children who had used RFW services within the 2 years prior to when the interviews were conducted were invited to participate in an interview. Semistructured interviews allow for detailed exploration of participants’ experiences and perceptions, including the ability to ask follow-up questions. Interview questions were provided to participants in advance of the interview so they had time to prepare their thoughts. The interview questions focused broadly on challenges and enablers of service engagement throughout a family’s service journey, with a substantial portion of the interview focusing specifically on experiences with telehealth. Questions were piloted with 2 individuals and refined prior to participant recruitment, resulting in minor changes to question wording.

### Data Collection

Interviews were conducted by a non-Indigenous female individual with extensive qualitative and mixed methods research experience who was employed as a postdoctoral researcher. Although the interviewer had presented the study outline earlier to many of the RFW service providers who later chose to participate, she had no prior relationship with caregiver interview participants. To facilitate rapport and relationship building, all interviews began with an invitation to ask the interviewer any questions about the study or the interviewer herself. All interviews took place online using Zoom teleconferencing software (Zoom Video Communications) and were audio recorded and transcribed using Zoom’s verbatim transcription service.

### Analysis

Once data saturation was reached, the interview data were analyzed thematically [19] using framework analysis [20], which includes the following 7 steps: (1) transcribing, (2) familiarizing oneself with the interview, (3) coding, (4) developing an analytical framework, (5) applying the analytical framework, (6) charting the data into a framework matrix, and (7) interpreting the data. Data were independently coded by 2 members of the research team, who identified emerging themes deductively using the Microsoft Word and Microsoft Excel software. Framework analysis allows for findings to be traced directly to primary data.

### Ethical Considerations

This study was approved by the University of Sydney Human Research Ethics Committee (2024/HE000682) and all participants provided written informed consent before participating in an interview. Interview participants were provided with an AUD $30 (US $24.50) grocery voucher in gratitude for their time.

## Results

### Participants

Semistructured interviews were conducted with 11 caregivers, all of whom were female and 81.8% (9/11) of whom were non-Indigenous, and 21 RFW service providers, including psychologists (n=3, 14.3%), social workers (n=4, 19%), occupational therapists (n=3, 14.3%), speech pathologists (n=6, 28.6%), pediatricians (n=2, 9.5%), and operations staff (eg, bookings or caregiver support services; n=3, 14.3%). Findings from the caregiver interviews are presented first, followed by service provider interview findings.

### Caregiver Findings

Three themes were identified within the caregiver interview data: (1) challenges with telehealth, (2) value of telehealth, and (3) settings for telehealth (Table 1). Some caregivers found telehealth to be a challenge, noting their children’s resistance to engaging with telehealth; distractions in the household setting; the inconvenience of the timing of telehealth appointments; and technological issues such as satellite internet, which is vulnerable to weather changes. Other caregivers found telehealth very helpful, noting as strengths their own and their children’s fluency with technology and the opportunity to access services that would otherwise be inaccessible. Finally, some caregivers recommended ensuring that the physical setting is conducive to telehealth.

**Table 1.** Caregiver interview findings regarding the challenges and value of telehealth.

Theme	Illustrative quotes
Challenges of telehealth	“We struggle with telehealth. [Child] just can’t. He struggles with social interaction as it is, let alone being face-to-face with someone on a computer. It just didn’t work for us. I don’t think there was anything that [Royal Far West] could do to make it better. They did try their best. But yeah.” [PDPF03] “And with the telehealth visits, at times I’d have the other kids. So, they’d be in the background watching TV. And then [oldest child] gets distracted. [And] the first was a week of telehealth visits. [...] We’ve only got satellite Internet. So, the pings have got to physically go up 30,000 Ks and back down. So, sometimes I’m still talking, and then someone else starts. Like, it doesn’t quite exactly work.” [PDPF02]
Value of telehealth	“I don’t see many challenges with telehealth because I’ve been using telehealth for a few years. And as someone who lives regionally, where services are so limited face-to-face, and I’m time poor. I work full time; I’m a single parent to two kids. So, I guess the general challenges would be, yes, you’re not seeing the person face-to-face, and that sort of stuff. But I don’t really see a challenge with telehealth. Had that [parenting] course been done any other way, I wouldn’t have been able to do it. Wouldn’t have mattered weekend, daytime, afternoon, I simply would not have been able to do it. So, I think it’s fantastic that we have a telehealth.” [CRPF01] “I’m very confident and capable with technology. So, I wasn’t apprehensive. I found no challenges with that. [...] [Child] has no challenges with digital tech, either.” [CRPF03] “I don’t remember there being any massive challenge [with telehealth]. I could see how someone a little bit more remote than me, or more rural than me could have internet issues, Wi-fi. Sometimes, if it’s really windy here, we’ll have trouble with our Wi-fi. But I think I was really lucky in a sense of I never really had any trouble like that.” [CRPF02]
Settings for telehealth	“Making sure the space is right. If you’re a family and you’re in a small house, like a lot of families have multiple people living in a house, it’s too distracting for a child. You need to make sure the space for the telehealth session to actually go ahead [is] quiet. Are they going to be able to focus?” [PDPF04]

### Service Provider Findings

Four main themes emerged from the service provider interview data: (1) the value of telehealth for rural and remote families, (2) implications of technology, (3) clinical challenges of telehealth, and (4) risk and safety (Table 2). Many service providers noted the value of telehealth, particularly in terms of its low cost to both families and RFW, facilitating access to care in the absence of other alternatives in local communities, and its demonstrable effectiveness for some disciplines. Others noted that some children respond really well over telehealth, whereas other children prefer face-to-face appointments. However, service provider interview respondents also noted several challenges with telehealth, including technological challenges for rural families, such as limited internet connectivity and difficulties accessing laptops or tablets for appointments.

**Table 2.** Service provider interview findings regarding the value and challenges of telehealth for rural and remote families.

Theme	Illustrative quotes
Value of telehealth for rural and remote families	“I think telehealth is amazing, because so many of those kids in rural, regional New South Wales, the waitlist is so long, or it’s so expensive, or they just don’t have any local services, or the local service is a two-hour drive away. So, telehealth is great in that we can offer them a service where they couldn’t get it otherwise.” [PDPS09] “The evidence, particularly for speech pathology, a lot of the evidence says [telehealth] is just as effective as in-person therapy. [...] So, I think it’s beneficial in terms of ease of access, cost, and we know that it’s just as effective. We can meet goals.” [PDPS13] “From an [occupational therapy] perspective, I was...kind of surprised at how well you can go on telehealth in terms of running different OT activities, chatting to parents, building relationships over telehealth has been amazing. And that’s the same for assessment as well.” [PDPS08]
Implications of technology	“The availability of technology. Some families might not have a laptop where they can sit down and have the zoom sessions and things like that. So that can be quite tricky, or they might not have good Internet at home, so that their availability of accessing telehealth can be tricky.” [PDPS08] “I think working on telehealth actually made me a much better [occupational therapist] in terms of me not being so reliant on equipment or games of those kind of things. Just thinking about, what can we do just with our bodies? Just being able to run an OT session with almost nothing. Very basic.” [CRPS06]
Clinical challenges of telehealth	“I find telecare incredibly challenging, for the social work role specifically.... I actually haven’t found telecare assessments that clinically valuable because we’re expected to do a [relatively] quick assessment. ...I think to build the rapport, to deep dive into your mental health, your relationship with your partner that you’re parenting with, or the reason why you’ve separated your co-parenting, what choices or strategies do you use in your parenting? All these pretty significant personal topics. To get there via telecare can feel really challenging. ...I just don’t ever think I get the same depth.” [PDPS03] “I do think telecare is a bit of a challenge. It certainly opens up doors in the sense of that means that we can check in with families, but it absolutely 100% does not replace a face-to-face assessment.” [PDPS15]
Risk and safety	“Challenges with telehealth and never knowing the full picture can put clinicians and the child at risk of child protection concerns—if child protection concerns are apparent [but] not able to be addressed, identified, and appropriately addressed.” [CRPS01]

Another challenge noted by service providers was the limited depth of clinical assessments possible for some disciplines such as social work. Some service providers also found standardized assessments difficult to complete via telehealth, noting that, at times, it is difficult to engage children on-screen for adequate periods. Service providers also commented on risk and safety concerns for children and their families when using telehealth, particularly in relation to gauging off-screen activities. As one service provider expressed, “I just don’t trust what I can’t see on screen” (PDPS03).

### Summary

In summary, although caregivers identified challenges, they also asserted the value of telehealth for multidisciplinary mental and developmental health assessments and treatment. Service providers reflected on both strengths and challenges associated with telehealth for multidisciplinary developmental and mental health assessment and treatment.

## Discussion

### Principal Findings

Caregivers and service providers articulated common themes when discussing the benefits and challenges of telehealth service delivery within a multidisciplinary pediatric developmental and mental health service for rural and remote children. Caregivers experienced challenges with telehealth yet valued it for its ability to facilitate access to otherwise inaccessible services. They also described the importance of ensuring that settings were conducive to telehealth assessment and treatment. Meanwhile, service providers reflected on the value of telehealth, particularly in terms of lower costs, easier access, and its demonstrable effectiveness for some clinical disciplines. Nevertheless, they also identified challenges with telehealth, including risk and safety; implications of technology; and depth of clinical assessments, particularly in relation to psychosocial evaluation. Importantly, while both caregivers and service providers expressed some concerns about telehealth, both groups shared an appreciation for how telehealth allows rural and remote children to access multidisciplinary developmental health services, especially given the dearth of appropriate services in rural and remote locations in Australia.

Cross-referencing caregiver and service provider perspectives on the challenges and value of telehealth for pediatric developmental and mental health reveals that multidisciplinary and multimodal telehealth can be an enabler of service engagement for rural and remote families along their care journey, particularly when used dynamically. For example, at RFW, telehealth is used for rapport building and service familiarization, diagnostic assessment, feedback, psychoeducation, therapy, care planning, progress review, and follow-up, as well as parenting programs. Thus, our findings suggest that telehealth can be effective for this range of activities when used to complement face-to-face consultations. Telehealth diagnostic assessment of complex developmental disorders elicited high levels of acceptance from caregivers and service providers despite limitations such as technical issues.

Our findings are consistent with those of international and Australian literature regarding telehealth in urban settings. For example, in an Australian metropolitan pediatric context, the use of telehealth in the diagnostic assessment of complex developmental disorders received high levels of acceptance from caregivers and service providers despite limitations similar to those observed in our study, such as technical issues, because it reduced costs, increased flexibility, and accommodated family needs [21]. Similarly, the international literature indicates that implementing telehealth in urban settings holds promise for greater accessibility of child mental health services [22], including pediatric psychiatry [23]. Our findings are also consistent with findings that telehealth supported increased rates of rural and remote patient satisfaction with allied health services such as occupational therapy and speech pathology [24].

### Implications

Our study suggests several implications and recommendations for policy and practice in the field of telehealth for child mental and developmental health assessment and treatment. First, rural and remote communities without access to face-to-face services should have access to telehealth services that incorporate family, educational, and societal factors impacting the child. This would empower children and families with psychosocial or neurodivergent complexity to seek care and optimize access to government rebates for care needs that depend on multidisciplinary clinical assessment. Second, to be effective, digital literacy and internet connectivity must be assured. Finally, service provision staff must be supported to deliver telehealth services in integrated and dynamic ways.

### Strengths and Limitations

One strength of our study is that it triangulates primary data from both caregiver and service provider interviews, facilitating comparisons of their experiences and perspectives. In addition, member checking of primary data before analysis, reflexivity throughout the analysis period, and an audit trail supported by the systematic stages of framework analysis strengthened the rigor and quality of data interpretation. However, this study had several limitations. For example, one potential limitation is self-selection bias because participants who voluntarily chose to take part represent only part of the client cohort. Similarly, caregiver interviewees were all biological mothers, omitting the perspectives of other caregivers (eg, fathers or grandparents). Another limitation relates to constraints on transferability given the relatively small sample sizes, as well as the fact that service clients (ie, children) were not engaged directly in the research. Despite these limitations, our qualitative findings provide important insights into the role of telehealth for rural and remote access to developmental and mental health services.

### Directions for Future Research

Future studies should explore rural and remote children’s experiences of telehealth and how it can inclusively engage and support neurodivergent or culturally diverse groups. Caregiver perspectives on unique aspects of telehealth clinician interactions, including supportive evaluation of child and family risk issues, could also be explored. Given the centrality of telehealth to rural and remote care, ongoing investigation of innovative telehealth neurodevelopmental assessment and therapeutic tools should be pursued, as well as comparison of care satisfaction, impact, and outcomes of telehealth vs hybrid telehealth and face-to-face care.

### Conclusions

Although there are challenges with telehealth, it remains a vital option within multidisciplinary services to improve access and outcomes for children in rural and remote communities who present with complex mental and developmental health needs. Telehealth can be used to deliver a range of clinical services and offers opportunities to develop new clinical approaches. Future work should explore approaches that allow for the adaptation of in-person assessments to telehealth and improve service provider skills in engaging children using telehealth. As technology improves, so should the accessibility of telehealth-delivered care.

## Abbreviations

ASD: autism spectrum disorder
RFW: Royal Far West
